# Cortical and subcortical gray matter changes in patients with chronic tinnitus sustaining after vestibular schwannoma surgery

**DOI:** 10.1038/s41598-021-87915-3

**Published:** 2021-04-16

**Authors:** Leonidas Trakolis, Benjamin Bender, Florian H. Ebner, Ulrike Ernemann, Marcos Tatagiba, Georgios Naros

**Affiliations:** 1 Department of Neurosurgery and Neurotechnology, Eberhard Karls University Hospital, Hoppe-Seyler-Straße 3, 72076 Tuebingen, Germany; 2 Department of Diagnostic and Interventional Neuroradiology, Eberhardt Karls University Hospital, Tuebingen, Germany; 3 Department of Neurosurgery, Alfried Krupp Hospital, Essen, Germany

**Keywords:** Experimental models of disease, Cortex

## Abstract

Tinnitus is attributed to partial sensory deafferentation resulting in a central maladaptive neuroplasticity. Unfortunately, the agent of deafferentation is usually unknown or irreversible. In patients with unilateral vestibular schwannoma (VS), however, the auditory nerve is affected by a benign tumor. Hence, removal of the tumor can cease the tinnitus. In turn, sustaining complaints after surgery indicate cortical neuroplasticity. The present study is a cross sectional study which aims to track cortical structural changes by surface-based morphometry in 46 VS patients with sustained (i.e. centralized) or ceased (i.e. peripheral) tinnitus after surgery. A volumetric analysis of cortical and subcortical gray matter (GM) anatomy was performed on preoperative high-resolution MRI and related to the presence of hearing impairment, pre- and/or postoperative tinnitus. Patients with sustained (i.e. chronic) tinnitus showed an increased GM volume of the bilateral caudate nucleus, the contralateral superior colliculus, the middle frontal and middle temporal gyrus, the fusiform gyrus as well as the ipsilateral pars orbitalis when compared to those patients in whom tinnitus ceased postoperatively. Chronic tinnitus in VS patients is associated with characteristic structural changes in frontal, temporal and subcortical areas. Notably, a significant GM change of the caudate nucleus was detected providing further support for the striatal gaiting model of tinnitus.

## Introduction

The current pathophysiological concept of tinnitus attributes spurious auditory signals after partial sensory deafferentation to the onset of the symptom^1–6^. After chronification, however, tinnitus perpetuation is theorized to depend on central maladaptive neuroplasticity as a consequence of the disturbed signal-to-noise ratio. These neuroplastic changes are thought to cause a neuronal hyperexcitability for the residual auditory input resulting in the subjective misperception^7–10^. To validate this theory of the pathophysiological origin of tinnitus, there is an increasing interest in magnetic resonance imaging (MRI) techniques, such as voxel-based (VBM) or surface-based (SBM) morphometry, to determine structural changes in the brain of tinnitus patients. Although a various number of studies using structural MRI have been performed to explore the etiology of tinnitus, the results are inconsistent and partially conflicting^11,12^. In addition to methodological and statistical drawbacks special attention has been drawn on the heterogeneity of the analyzed patient cohorts which could have affected the MR findings^12^. Along this line, further differentiation of tinnitus patients have shown subtype-specific cortical changes^13,14^. Unfortunately, for most tinnitus patients the agent of sensory deafferentation is either unknown or irreversible impeding causal therapy^8,10^. In patients with a unilateral vestibular schwannoma (VS), however, the auditory nerve is affected by a benign vestibular nerve tumor^15^. Today, the continuous progress in surgical techniques enables a gentle tumor removal with functional preservation of hearing nerve in a large number of patients^16–18^. Resulting from this technical progress, VS-associated tinnitus ceases in one third of the patients after surgical removal of the tumor^19–21^. However, tumour removal is expected to terminate tinnitus only in the cases in which peripheral source is the underlying cause^19^. In turn, we hypothesize that any sustaining complaints after VS surgery are attributed to a centralization (i.e. cortical neuroplasticity) of the tinnitus^19^. However, all MRI studies, with the exception of three studies^22–24^, evaluating tinnitus-related brain structure have excluded patients with anatomical causes of tinnitus, such as VS (for review see Scott-Wittenborn et al. 2017). However, there is no study analyzing explicitly patients with VS-associated tinnitus, who represent a relatively homogenous patient cohort^15^. The present study aims to track structural changes of gray matter depending on tinnitus outcome after VS surgery.

## Results

### Audiovestibular symptoms of the patient cohort

This retrospective cross section study enrolled 46 consecutive patients with unilateral sporadic VS. Basic patient characteristics are shown in Table 1. Preoperatively, 67% patients had a functional hearing (GR1/2). None of the patients suffered from deafness. 57% patients suffered from preoperative tinnitus (preopTN) which disappeared in 62% of the cases after surgical VS removal. 22% patients suffered from a preoperative tinnitus which then sustained for at least three months after surgery. Overall, there were 26% patients suffering from postoperative tinnitus (postopTN), indicating 2 new-onset tinnitus. 63% patients suffered from vestibulopathy (i.e. vertigo or dizziness), preoperatively. Patients with preoperative tinnitus where more likely to suffer from vestibulopathy (X^2^ = 4.95, p = 0.26) and of the 10 patients with chronic tinnitus, only 2 did not suffer from a vestibulopathy.

**Table 1:** Summarizing the patient characteristics.

Age	47.5 ± 11.6 years [24–75]
**Gender**	
Female	50.0% (23/46)
Male	50.0% (23/46)
**Lesion size**	
T1/2	17.4% ( 8/46)
T3	50.0% (23/46)
T4	32.6% (15/46)
**Lesion side**	
Left	34.8% (16/46)
Right	65.2% (30/46)
Preoperative tinnitus	56.5% (26/46)
**Side of preoperative tinnitus**	
Left	34.8% (16/46)
Right	65.2% (30/46)
**Postoperative tinnitus**	
Overall	26.1% (12/46)
Sustained (i.e. chronic) tinnitus	21.7% (10/46)
New-onset tinnitus	4.4% (2/46)
**Preoperative hearing**	
Functional hearing (GR°I/II)	67.4% (31/46)
Non-functional hearing (GR°III/IV)	32.6% (15/46)
Deaf (GR°V)	0% ( 0/46)
**Preoperative acoustic evoked potentials**	
I	15.2% (7 /46)
II	34.8% (16/46)
III	10.9% ( 5/46)
IV	15.2% ( 7/46)
V	23.9% (11/46)
Preoperative dizziness and vertigo	63.0% (29/46)
**Preoperative facial score**	
H&B° I	97.8% (45/46)
H&B°II	2.2% ( 1/46)

### Volumetric changes in patients with sustained VS-associated tinnitus after surgery

**Figure 1:**
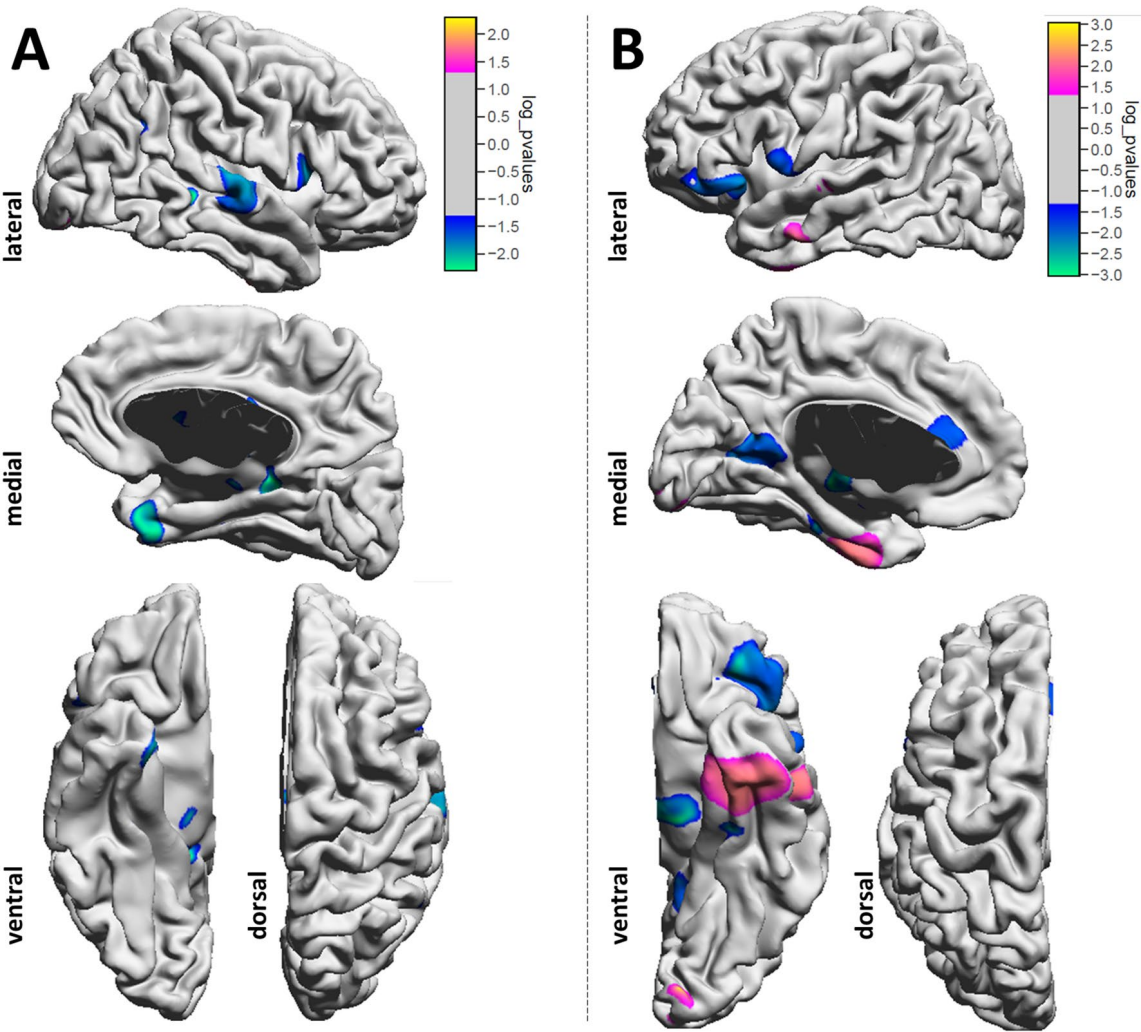
Whole-brain vertex-based statistical maps (corrected for TIV) indicated volumetric changes covering ipsilesional (**A**) and contralesional (**B**) frontal (e.g., opercular part of inferior frontal gyrus), temporal (e.g., medial temporal gyrus, fusiform gyrus) and mesial (e.g., cingulate gyrus and precuneus) structures. Notably, p value is not corrected for multiple comparisons. Results did not withstand FDR-based multiple comparison correction.

**Figure 2:**
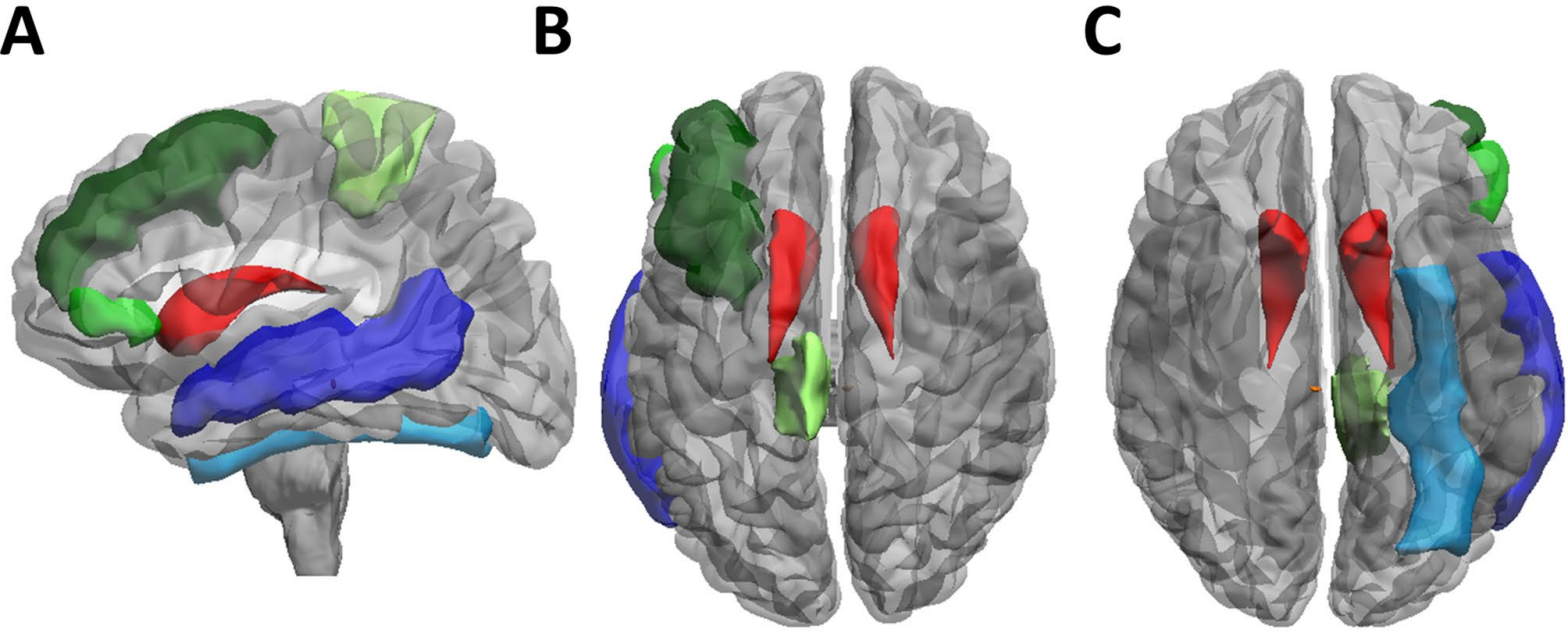
Anatomical localization of the significant volumetric changes in the ROI-based analysis shown in (**A**) left-lateral (i.e., ipsilesional), (**B**) dorsal and (**C**) ventral view. For the temporal cortex, a significant GM increase of the fusiform gyrus (light blue) and of the medial temporal gyrus (dark blue) in patients with sustained tinnitus after VS surgery. For the frontal cortex, there was an GM increase in the contralateral medial frontal gyrus (dark green) and the pars orbitalis of the inferior frontal gyrus (light green) as well as a significant GM decrease in the contralateral paracentral lobule (lime green). Finally, a significant GM increase of the ipsilateral superior colliculus (orange) in the bilateral caudate nucleus was detected (red).

A total of 10/46 patients (22%) who suffered from a preoperative tinnitus reported postoperative tinnitus that sustained for at least three months. These latter patients are considered to suffer from chronic tinnitus after centralization making neuroplastic cortical changes more likely. To evaluate volumetric gray matter (GM) changes related to sustained VS-associated tinnitus (susTN), we performed a surface-based morphometry on patients’ individual structural MRI. Whole-brain vertex-based statistical maps (corrected for TIV) indicated volumetric changes covering ipsilesional and contralesional frontal (e.g., opercular part of inferior frontal gyrus), temporal (e.g., medial temporal gyrus, fusiform gyrus) and mesial (e.g., cingulate gyrus and precuneus) structures (Fig. 1). However, results did not withstand FDR-based multiple comparison correction. Subsequently, we performed a ROI-based analysis (Table 2). There was a significant multivariate effect of susTN on GM volumetry (F_(44,1)_ = 987.50, p = 0.025; Wilks’ Λ < 0.001) was detected. The results of the FDR-corrected follow-up ANOVAs are reported according to their location, i.e. temporal, frontal or subcortical (Fig. 2).

**Table 2:** Region-of-interest (ROI).

ROI	Contralat. ROI-ID	Ipsilat. ROI-ID	ROI	Contralat. ROI-ID	Ipsilat. ROI-ID
Superior frontal gyrus	121	120	Inferior temporal gyrus	329	328
Middle frontal gyrus	131	130	Fusiforme gyrus	331	330
Pars opercularis	143	142	Parahippocampal gyrus	343	342
Pars triangularis	145	144	Hippocampus	345	344
Pars orbitalis	147	146	Amygdala	347	346
PRE-central gyrus	151	150	Superior occipital gyrus	423	422
Transvers frontal gyrus	163	162	Middle occipital gyrus	425	424
Gyrus rectus	165	164	Inferior occipital gyrus	443	442
Middle orbito-frontal gyrus	167	166	Lingual gyrus	445	444
Anterior orbito-frontal gyrus	169	168	Cuneus	447	446
Posterior orbito-frontal gyrus	171	170	Insula	501	500
Lateral orbitofrontal gyrus	173	172	Caudate nucleus	613	612
Paracentral lobule	183	182	Putamen	615	614
Cingulate gyrus	185	184	Globus pallidus	617	616
Subcallosal gyrus	187	186	Nucleus accumbens	621	620
Post-central gyrus	223	222	Claustrum	631	630
Supramarginal gyrus	225	224	Thalamus	641	640
Angular gyrus	227	226	Basal forebrain	651	650
Superior parietal gyrus	229	228	Lateral geniculate nucleus	661	660
Pre-cuneus	243	242	Medial geniculate nucleus	663	662
Temporal pole	311	310	Superior colliculus	671	670
Superior temporal gyrus	323	322	Inferior collicullus	681	680
Heschl’s gyrus	325	324	Mamillary body	691	690
Middle temporal gyrus	327	326	Brainstem	800	
			Cerebellum	900

#### Temporal changes

**Figure 3:**
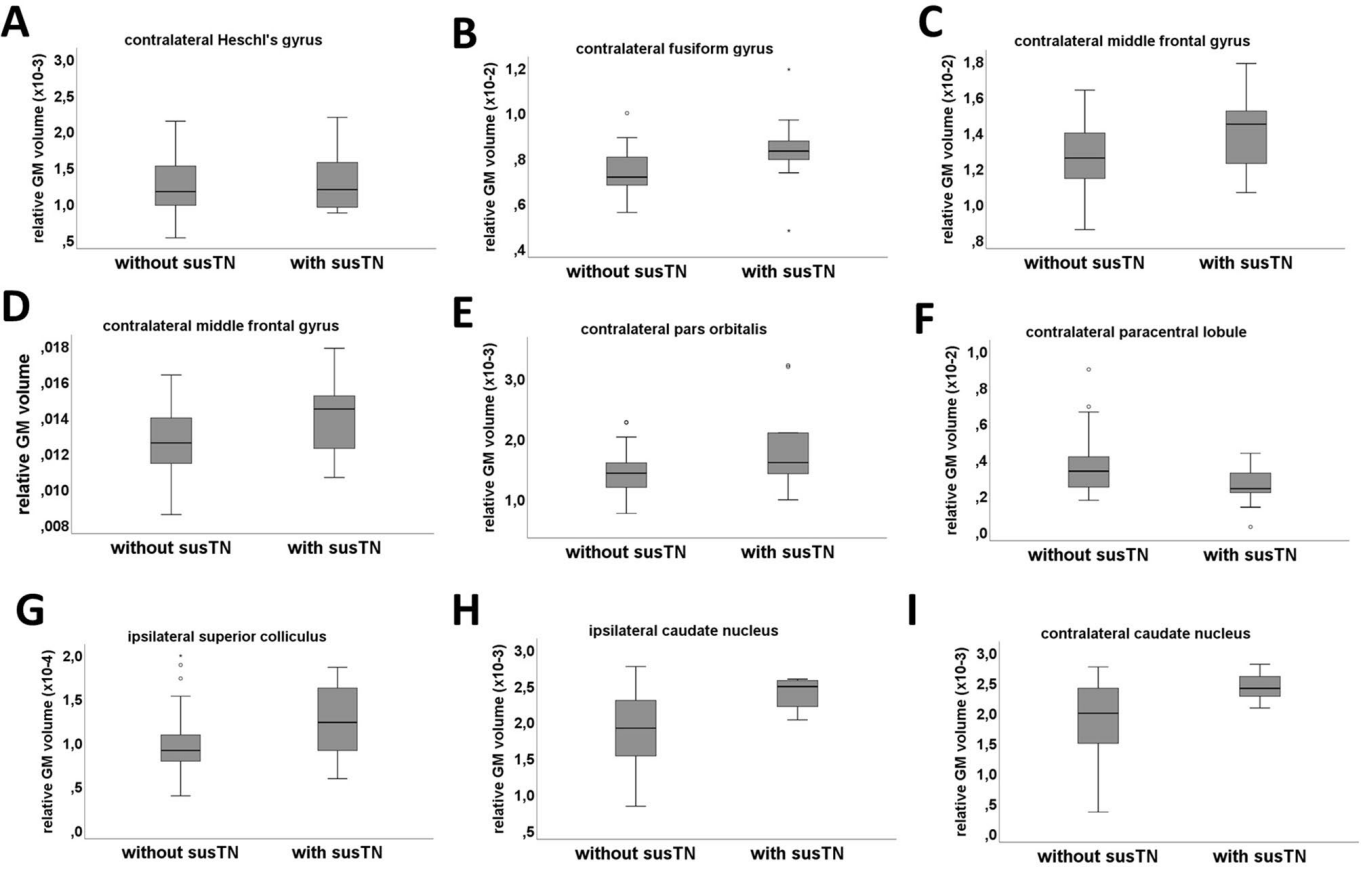
There was no volumetric change of the contralateral Heschl’s gyrus (**A**). However, there were significant GM changes in VS-associated tinnitus for the contralateral fusiform gyrus (**B**), the contralateral middle temporal gyrus (**C**), the contralateral medial gyrus (**D**), the contralateral pars orbitalis (**E**), the contralateral paracentral lobule (**F**), the ipsilateral superior colliculus (**G**) and the ipsilateral (**H**) as well as contralateral caudate nucleus (**I**). Data is shown in boxplot (*thick line*: median; *box width*: first and third quartiles; *whiskers*: minimum and maximum values; *circle and asterisks*: outliers and extremes).

Patients with chronic tinnitus did not show any GM changes of the contralateral Heschl’s gyrus (1.3 × 10^–3^ ± 0.6 × 10^–3^ and 1.3 × 10^–3^ ± 1.5 × 10^–3^, F_(1,44)_ = 0.05, p = 0.891; Fig. 3A). However, there was a GM increase of the contralateral fusiform gyrus (7.3 × 10^–3^ ± 0.9 × 10^–3^ and 8.3 × 10^–3^ ± 1.8 × 10^–3^, F_(1,44)_ = 6.02, p = 0.018; Fig. 3B) and the contralateral middle temporal gyrus (12.9 × 10^–3^ ± 0.2 × 10^–3^ and 14.7 × 10^–3^ ± 0.2 × 10^–3^, F_(1,44)_ = 4.59, p = 0.038; Fig. 3C).

#### Frontal changes

At the same time, VS patients with chronic tinnitus show an increase in the volume of contralateral medial frontal gyrus (12.5 × 10^–3^ ± 1.8 × 10^–3^ and 13.9 × 10^–3^ ± 2.1 × 10^–3^, F_(1,44)_ = 4.76, p = 0.035; Fig. 3D) and pars orbitalis (1.4 × 10^–3^ ± 0.4 × 10^–3^ and 1.9 × 10^–3^ ± 0.8 × 10^–3^, F_(1,44)_ = 6.87, p = 0.012; Fig. 3E). However, there was a significant GM decrease in the contralateral paracentral lobule (3.5 × 10^–3^ ± 1.5 × 10^–3^ and 2.4 × 10^–3^ ± 1.1 × 10^–3^, F_(1,44)_ = 4.57, p = 0.038; Fig. 3F).

#### Subcortical changes

Finally, there was a significant GM increase of the ipsilateral superior colliculus (9.6 × 10^–5^ ± 3.8 × 10^–5^ and 12.5 × 10^–5^ ± 4.3 × 10^–5^ , F_(1,44)_ = 4.40, p = 0.042; Fig. 3G) as well as the and ipsilateral (1.9 × 10^–3^ ± 0.5 × 10^–3^ and 2.3 × 10^–3^ ± 0.2 × 10^–3^, F_(1,44)_ = 9.11, p = 0.004; Fig. 3H) and the contralateral caudate nucleus (1.8 × 10^–3^ ± 0.6 × 10^–3^ and 2.4 × 10^–3^ ± 0.2 × 10^–3^, F_(1,44)_ = 8.03, p = 0.007; Fig. 3I). The additional Student’s t-test analysis certified the effect of chronic tinnitus on the bilateral caudate nucleus (p = 0.012 and p = 0.003, Student’s t-test).

### Volumetric changes in relation to preoperative hearing impairment

**Figure 4:**
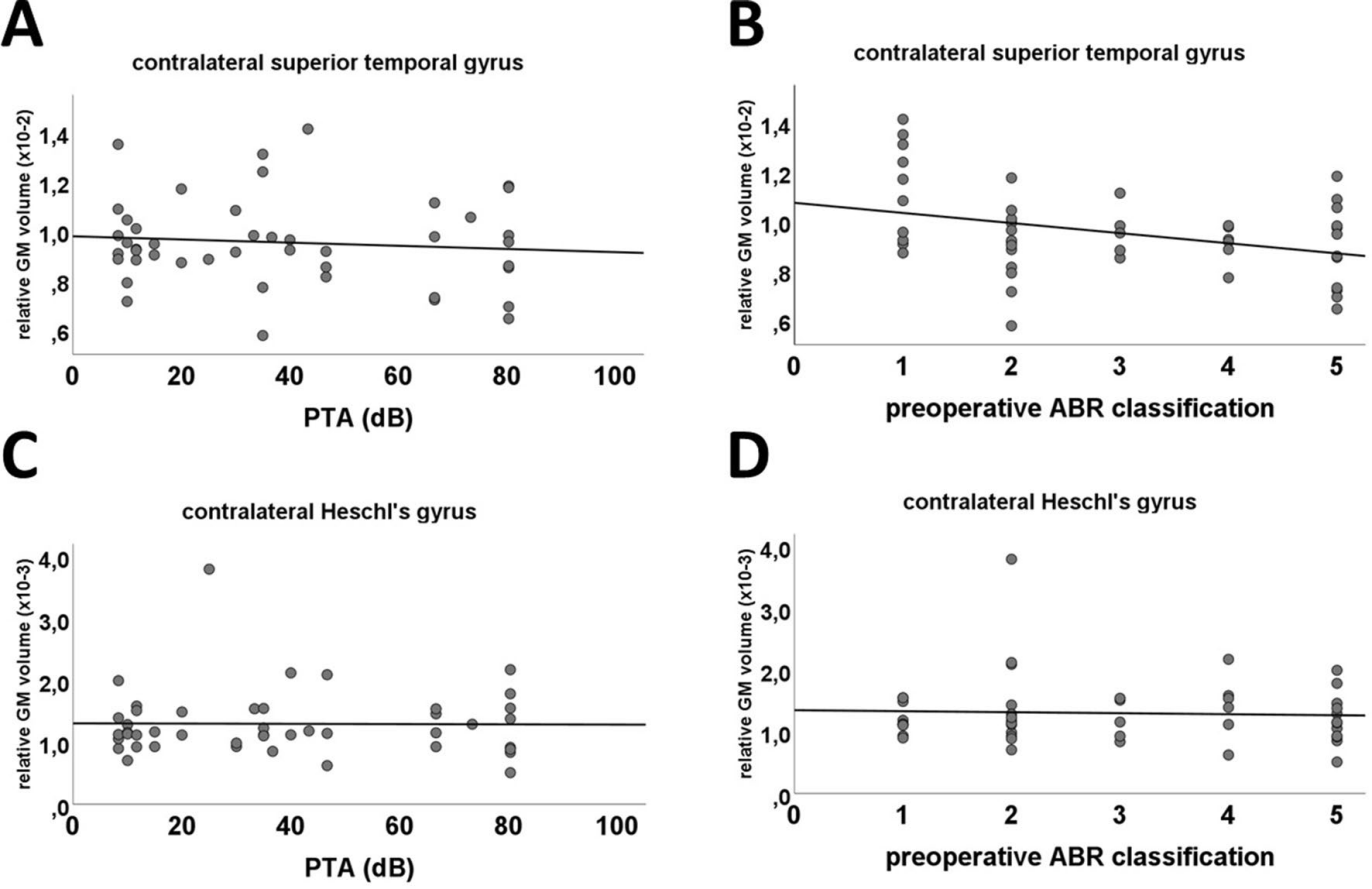
Correlation analysis showed no significant correlation between the relative GM volume of the contralateral superior temporal gyrus (**A,B**) or the contralateral Heschl’s Gyrus (**C,D**) to the preoperative hearing loss as measured by the PTA (**A,C**). However, there was a significant negative correlation between the preoperative AEP measurement and the GM volume of the contralateral superior temporal gyrus (**B**). In contfrast, there was no relationship between the AEP and the volume of the Heschl’s gyrus (**D**).

A multivariate analysis of variance (MANOVA) was applied to evaluate the effect of preoperative hearing impairment (preopHI) on the GM volume of 96 cortical and subcortical brain regions which failed statistical significance (F_(44,1)_ = 145.88, p = 0.066; Wilks’ Λ < 0.001). In line, there was no correlation between the GM volume of neither the contralateral superior temporal gyrus (r = − 0.1, p = 0.533; Pearson’s) nor the contralateral Heschl’s gyrus (r = − 0.01, p = 0.950; Pearson’s) to the patient’s preoperative hearing loss as measured by the PTA (Fig. 4A,C). However, when using the preoperative ABR measurement there was a significant negative correlation to the GM volume for the contralateral superior temporal gyrus (r = − 0.30, p = 0.041; Spearman’s; Fig. 4B). Nevertheless, we could not find a relationship between the GM volume of the contralateral Heschl’s Gyrus and the preoperative AEP measurements (r = − 0.01, p = 0.938; Spearman’s; Fig. 4D).

Notably, neither preopTN (F_(44,1)_ = 1.17, p = 0.640; Wilks’ Λ = 0.019) nor the patients’ gender (F_(44,1)_ = 3.10, p = 0.427; Wilks’ Λ = 0.007) did affect ROI volumes. Additionally, there was no correlation between patients’ TIV and age (r = − 0.218, p = 0.146; Pearsons’).

## Discussion

The inconsistent and partially conflicting results of the hitherto existing morphometry studies are attributed to methodological and statistical drawbacks as well as the heterogeneity of the analyzed patients^11,12^. The present study aimed to address these problems by concentrating on a -to our opinion- more homogenous patient cohort with VS-associated tinnitus^15,19^. The unique characteristic of this patient group lies in the fact that surgery nowadays is able to remove peripheral tinnitus source (i.e. tumor), potentially without causing new harm to the cochlear nerve^16–18^. This technique, in turn, is ceasing tinnitus postoperatively in a large number of the patients^19–21^. Sustained tinnitus after VS surgery could indicate a centralization (i.e. cortical neuroplasticity) of the tinnitus^19^. In line with this hypothesis, the present study found significant volumetric changes only in patients with sustained tinnitus covering temporal, frontal and the caudate nucleus but not in patients whose tinnitus ceased after surgery.

In contrast to other studies, we found no significant changes in GM volume due to the preoperative hearing impairment^25,26^. However, there was a significant negative correlation between the preoperative AEP measurement and the GM volume of the contralateral superior temporal gyrus. In contrast, there was no relationship between the AEP and the volume of the Heschl’s gyrus. These results are presented in Fig. 3.

Notably, as one of the few volumetric studies handling VS patients exclusively, Wang et al.^25^ showed a positive correlation between hearing loss and the decrease in the cortical thickness of the superior temporal gyrus and Heschl’s gyrus. On the other hand, Profant et al.^27^ reported a rather small effect of hearing loss on the auditory cortex. The present study could partially confirm these findings, however, likely attributable to the composition of the patient cohort, in which a great number of the analysed patients had good or at least functional hearing, whereas no patient was suffering from hearing loss. Considering the limited sample size, this could have influenced the present results.

In good agreement with other studies, our analysis provide additional evidence for an increase of the medial temporal gyrus in patients with persistent tinnitus^24,28–32^ which has implicated the medial temporal lobe as a final common pathway for all tinnitus patients^33^. Same results were presented from patients with chronic tinnitus and unilateral hearing loss as well. Although the fusiform gyrus is not yet associated with tinnitus, many reports relate it to speech processing and tone recognition^34,35^. We hypothesize, that VS patients might need more effort to recognise and process the sound due to the VS-associated tinnitus and hearing impairment. On the other side, it is well known that the fusiform gyrus is affected in patients with vestibulopathy. As 8/10 patients with sustained tinnitus have been suffering from preoperative vertigo and/or dizziness, the detected changes might be related to the preoperative vestibulopathy^32^.

Most detected volumetric changes are covering a tinnitus-related fronto-temporal network including auditory and limbic-associated areas that have been described in prior studies^36^. Surprisingly, our analysis provides evidence for a volume reduction of the paracentral lobule. Volumetric changes in this region associated to tinnitus have been described before^13^. Positron emission tomography, however, have shown an increased activity of this region in tinnitus^28,37^. Nevertheless, the involvement of the paracentral lobule in tinnitus pathophysiology is still unclear. Besides, the present study revealed a volume increase of the contralateral medial frontal gyrus, the location of the ventromedial (vmPFC) and dorsomedial prefrontal cortex (dmPFC). However, in contrast to our findings previous studies have shown a volume reduction^38–41^ correlating with perceptual loudness and awareness of the tinnitus sensation^40^. One possible explanation for this discrepancy might be the duration since the onset of tinnitus. While the previous studies evaluated patients suffering from tinnitus for several years or even decades, VS patients are usually treated surgically in a rather short time. In fact, concentrating on the patients with a duration since tinnitus onset < 5 year, vmPFC surface area and dmPFC curvature show a positive correlation to the tinnitus duration^40^. Initially, tinnitus is supposed to lead to a hyperactivity of the associated regions^42–44^. Chronic hyperactivity could, however, generate excitotoxic levels of glutamate receptor activation^45^. We hypothesize that after an initial volume increase of the medial frontal cortex prolonged exposure results in a cell death and successive volume reduction. The same pathophysiological concept is suggested for the volume increase of the pars orbitalis of the inferior frontal gyrus which is also in conflict to the volume reduction which was found in previous studies^22^.

Interestingly, the present study depicted volumetric changes in the tectum and the basal ganglia. Previous studies might have been unable to detect these changes as VBM is not optimal for assessing subcortical structures^40,46^. Although the connection of inferior colliculus in the auditory pathway through the temporal role and its abnormal activation in tinnitus is already shown^47–52^, those of the superior colliculus remains to be elucidated. Animal and human research has shown a relation of the superior colliculus activation to sound perception and its involvement in auditory pathways^47,53–56^. Furthermore, it is known that the deeper layers of superior colliculus receive auditory information from the inferior colliculus, in order to form audio-visual integration of speech^57^. In light of this, we observed a GM increase of the superior colliculus. Unfortunately, changes of the superior and inferior colliculus did not reach statistical significance. However, the interpretation of this result must consider the technical limitation of SBM with thin gyral stalks^58^. Finally, one of the most robust findings in our data, depicted by all three statistical evaluations, was a bilateral increase of GM volume of the caudate nucleus. The basal ganglia, in particular the caudate nucleus, is usually not in the principal focus of tinnitus research. However, there is evidence for an increased connectivity of the basal ganglia in chronic tinnitus patients^59,60^. This received further support by the recent findings that there is an increased connectivity between the caudate nucleus and the cortex in chronic tinnitus patients^61^. Similar to our results with changes in the limbic system and the bilateral peri-auditory regions in patients with chronic tinnitus were delivered from Meyer et al.^62^. Comparable to this study, volumetric changes were independent of age, hearing loss and sex^62^.

The caudate nucleus is strongly interconnected with the frontal limbic system^61,63–65^, including vmPFC which is related to the perception of tinnitus and is thus part of the gating system^36^. A recent development is the striatal gating model which hypothesizes the caudate nucleus to act as a gating mechanism for tinnitus awareness^66^. This hypothesis was initially based on the observation that direct stimulation of the dorsal striatal area LC, a locus of nucleus caudatus located at the junction of the head and body of the caudate nucleus, during deep brain stimulation (DBS) surgery in patients with movement disorders modulates auditory phantom loudness^67^ and triggers auditory phantom percepts in patients with hearing loss^66^. Furthermore, vascular infarction of area LC results in enduring tinnitus loudness suppression^68^. However, the exact physiological mechanism is unclear. Due to its interconnection to both the auditory cortex and the frontal limbic system (e.g. vmPFC)^61,64,65^ the caudate nucleus could act as a switch for auditory phantom representations to reach perceptual awareness and define its severity. In fact, a recent study has been able to show an increased functional connectivity between the left caudate nucleus and the left Heschl’s Gyrus as well as the left and right Heschl’s Gyrus and the left caudate nucleus in patients with bothersome tinnitus and single-sided deafness^65^. Notably, these connections correlated with the difficulty to relax due to the tinnitus and with the sense of reduced control over the tinnitus^65^. Furthermore tinnitus is shown to enhance the functional connectivity between the temporal cortex and the limbic system which explains the volumetric changes we found in these areas^27,69^. The present study is the first volumetric study to show GM changes in the caudate nucleus which could be a structural correlate of the augmented functional interactions of the caudate nucleus and cortical hearing and non-hearing centres in tinnitus.

This study comes however with some limitations. Although the SBM approach is supposed to be robust to different field strengths, and different scanner specifications^70^, combining scans from multiple centres introduces a bias. However, as the study design is balanced, scanner differences affect all patients and are not specific to one clinical subgroup. Although having a rather large patient cohort in comparison to related studies^12^, subgroup analysis within the present study is limiting the sample size of each group. Although sample size calculations for neuroimaging studies are challenging^12^, studies with larger patient cohorts are needed to verify structural changes in VS-associated tinnitus patients. A major limitation of the study is the dichotomization of the patients’ tinnitus complaints. Although advantageous for the statistics, there was available systematic data on the tinnitus severity (e.g. Tinnitus Handicap Inventory) due to the retrospective design of the study. Finally, methodological aspects should be discussed. Several software packages offer automated voxel-/vertex-based morphometry solutions (e.g., Brainsuite, Freesurfer, SPM). While most of the available studies apply Freesurfer or SPM^12^, the present study was performed with the Brainsuite software. Thus, results are not directly comparable to the available data^12^. However, Brainsuite is known to provide results of similar accuracy^71,72^ and might be more suitable for subcortical structures^62,73–75^.

To sum up, up to date volumetric studies in VS-associated tinnitus could help trace the pathophysiology of this disease due to the unique possibility of causal therapy in these patients. Hence, the present study represents, to our knowledge, the first volumetric study showing GM changes in VS-associated tinnitus. In fact, sustained tinnitus after VS surgery was associated with structural changes in frontal and temporal regions comparably to non-VS tinnitus studies. However, although using a rather homogeneous patient cohort the present study could not resolve all discrepancies which are obviously inherent in comparable studies. Nevertheless, it is noteworthy, that for the first time a significant GM volume increase was detected for the caudate nucleus providing further support for the striatal gaiting model in tinnitus. This knowledge provides further insight in the pathophysiology of tinnitus and might help to predict tinnitus outcome after VS surgery which improve preoperative counselling and might affect surgical strategy. Further prospective studies with a larger patient cohort and standardized MRI-scanner protocols should be performed, which will enlighten us about the VS-associated tinnitus development and persistence.

## Methods

### Patients

This retrospective cross section study enrolled 46 consecutive patients with unilateral sporadic VS who underwent a neurosurgical VS removal in the Neurosurgical Department of the University of Tuebingen, Germany between January 2008 and January 2015. The inclusion criteria covered an age range of 18–80 years old and the availability of preoperative high-resolution magnetic resonance imaging (MRI) T1 sequences, as the later is not part of the usual neuroradiological workup of VS patients. All patients received clinical evaluations of VS-associated symptoms (i.e. tinnitus and hearing impairment) prior to and three months after surgery. The principles of the surgical procedure were unchanged throughout all patients^17^. Patient characteristics are shown in Table 1. 26/46 patients (57%) suffered from preoperative tinnitus which disappeared in 16/26 (62%) of the cases. This study was approved by the ethics committee of the Eberhard Karls University Tuebingen (registration no. 513/2017B02). All participants gave informed consent. All methods were performed in accordance with the guidelines and regulations.

### Clinical evaluation

All patients underwent a thorough clinical evaluation of VS-associated symptoms (i.e. hearing impairment, tinnitus, dizziness, balance problems, gait disturbance, headache, facial dysesthesia and/or palsy, swallowing difficulties, nausea, vomiting) by a semi-structured interview by experienced neurosurgeons. Finally, the presence of ipsilateral tinnitus symptoms was dichotomized for statistical analysis (0: no tinnitus, TN-; 1: tinnitus present, TN +). Hearing impairment was classified according to the Gardner & Robertson (GR) scale^76^ based on the results of the pure tone audiometry (PTA) and speech discrimination (SDS) resulting in five classes: GR 1 (good, PTA 0–30 dB and SDS 70–100%), GR 2 (serviceable, PTA 31–50 dB and SDS 50–69%), GR 3 (non-serviceable, PTA 51–90 dB and SDS 5–49%), GR 4 (poor, PTA 51–90 dB and SDS 1–4%), GR 5 (deaf, PTA 0 dB and SDS 0%). According to previous publication, the GR score was reclassified in (i) functional hearing (GR1 and GR2), (ii) non-functional hearing (GR3 and GR4) and (iii) no hearing (GR5)^19,20,77^. Additionally, hearing function was classified based on the presence or absence of waves I, III and V in the auditory brainstem response (ABR) examination performed before surgery: Group I (Waves I, III and V are present and the latency I-III is normal or slightly increased), Group II (Waves I, III and V are present and the latency I-III is pathologically increased > 2.66 ms), Group III (Wave III is lost but Waves I and V are present), Group IV (only wave I or wave 5 is present) and Group V (all waves are lost). VS tumor size was graded according to Koos classification^78^ into 4 classes: T1 (purely intrameatal), T2 (intra- and extrameatal), T3 (filling the cerebellopontine cistern), T4 (compressing the brain stem).

### MRI acquisition and processing

MRI data was acquired for preclinical diagnostic purposes on different MR-tomographs (43 cases with 1.5 T, 3 cases with 3 T). However, only patients with preoperative high-resolution 3D T1-weighted sequences in the sagittal plane with Gadolinium-based contrast agents were enrolled in the study (isovoxel of 0.5–1.0 mm in 36 patients; isovoxel of 1.2–2.2 mm in 10 patients). To ensure high MR quality (e.g. from movement artifacts), all images were carefully inspected.

### Volumetric analysis

**Figure 5:**
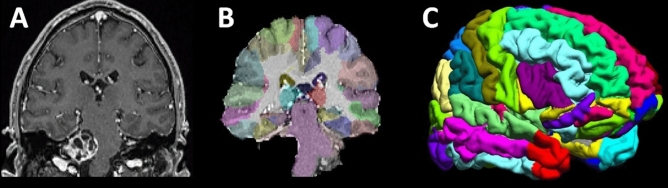
**(A)** Exemplary MR image of a patient with a T3 vestibular schwannoma of the right side. Data processing included skull striping and brain extraction process followed by the automated brain atlas registration **(B,C)**.

MRI data was flipped according to the affected AN (i.e., right hemisphere represents ipsilateral to the tumor) with the MRIcron software (http://www.nitrc.org/projects/mricron). Subsequent analysis steps were performed by the Brainsuite software package for SBM (http://www.brainsuite.org)^73^. In brief, Brainsuite performs a sequence of image analysis steps including skull and scalp removal, nonuniformity correction, tissue classification, topology correction, and surface generation to produce triangular surface mesh models of the inner and outer boundaries of the cerebral cortex. Next, the surfaces for each subject were registered to a reference atlas surface using Brainsuite's surface/volume registration software (SVReg, http://brainsuite.org/processing/svreg/)^79–81^. The SVReg-results were proofed manually to ensure proper segmentation and surface/volume registration (see Fig. 5). This results in a spatial alignment of the white/gray matter (WM/GM) cortical surfaces across all subjects. The GM volume of each region-of-interest (ROI) was averaged and used for ROI-based analysis (Table 2). For, vertex-vice (i.e., whole-brain) analysis data was smoothed using a 2.5‐mm kernel compensating for registration inaccuracies.

The GM volume of each ROI was normalized by division of the total cortical (GM + WM) volume^82,83^ in order to correct for global effects such as head size, age and gender^84,85^. Most commonly, an estimate of intracranial volume is including the cerebrospinal fluid (CSF + GM + WM)^86–89^. In the present study, however, CSF volume, in contrast to GM and WM, was not normally distributed (p = 0.023, Kolgomorov-Smirnov-Test) and was consequently not included in the normalization process. This could be explained by the fact that vestibular schwannomas affect CSF circulation by obstruction or malreabsorption^90,91^. Finally, the presence of tinnitus did not affect CSF volume in the present study (p > 0.756, Kruskal–Wallis).

### Statistics

All statistical tests were performed using R (http://www.r-project.org) and SPSS (IBM SPSS Statistics for Windows, Version 25.0. Armonk, NY: IBM Corp.). For whole-brain analysis, GM surface was analyzed with a General Linear Model (http://brainsuite.org/bss/) to test for the effect of susTN. TIV was included in the model as covariate. Vertex-based *p* values were corrected using a false discovery rate correction (FDR < 0.05) based on the Benjamini and Hochberg (BH) procedure^92^. For ROI-based analysis, separated multivariate analysis of variance (MANOVA) tests were applied to evaluate the impact of preoperative (preopTN) and sustained/chronic tinnitus (susTN) as well as the preoperative hearing impairment (preopHI) on the normalized ROI volumes. Given the known dependency between preopTN, susTN and preopHI^19^, including these factors in a single MANOVA could bias the results due to multicollinearity. Multivariate outlier in ROI data were excluded using Mahalanobis Distance. MANOVA was follow-up by univariate ANOVAs. Resulting p-values were corrected for multiple comparisons based on FDR (< 0.05). Correlation analysis were based on Pearson’s correlation. Data are shown as mean ± standard deviation (SD). Statistical significance was considered with p < 0.05 for each frequentist statistical test. This study followed the STROBE (STrengthening the Reporting of OBservational studies in Epidemiology) checklist.

## Data Availability

The dataset used and analyzed in this study is available from the corresponding author upon request.

## Abbreviations

VS: Vestibular schwannoma
VBM: Voxel-based morphometry
MRI: Magnetic resonance imaging
PET: Positron emission tomography
SBM: Surface-based morphometry
CSF: Cerebrospinal fluid
GM: Gray matter
WM: White matter
